# Polyelectrolyte Hydrogel Platforms for the Delivery of Antidepressant Drugs

**DOI:** 10.3390/gels2040024

**Published:** 2016-09-27

**Authors:** Mario Casolaro, Ilaria Casolaro

**Affiliations:** 1 Department of Biotechnology, Chemistry and Pharmacy, University of Siena, Via Aldo Moro 2, Siena 53100, Italy; 2 Psychiatry Resident, University of Siena, Siena 53100, Italy; casolaro.ilaria@gmail.com

**Keywords:** stimuli-responsive hydrogels, polyelectrolytes, pulsatile drug release, paroxetine, duloxetine

## Abstract

Some vinyl hydrogels containing α-amino acid residues (l-phenylalanine, l-valine) were used as polyelectrolyte platforms for the evaluation of the controlled release of two antidepressants (paroxetine and duloxetine). The closer acidity constant (pKa) values of the two drugs show a closer release profile in physiological phosphate buffered saline (PBS) buffer (pH 7.40) and for long periods of time. The great electrostatic interaction forces between the COO^−^ group of the hydrogel and the protonated secondary amino nitrogen of the drug are the main factor improving the release kinetics; this release was found to be slower compared to that of two structurally related drugs bearing the tertiary amino nitrogen atom (citalopram and trazodone). Moreover, at the lower value of pH 4.60, paroxetine showed a flatter release profile from the hydrogel containing the l-phenylalanine residues that, after six days, is half of that shown by duloxetine. Further effects due to steric and hydrophobic interactions may contribute to the different release profile. A further stimulation with alternating magnetic fields (AMF) of low frequency (20 kHz/50 W) enhanced the release of the drug at pH 7.40 from the hydrogel containing magnetic nanoparticles. Both AMF and PBS solution at pH 7.40 were used to trigger the ‘on-demand’ pulsatile paroxetine release from the nanocomposite hydrogel.

## 1. Introduction

Hydrogels are very promising soft materials as they offer the possibility of various applications, especially in future biomedicine and in the delivery of drugs [1,2,3,4,5,6,7]. They mainly consist of a macromolecular network capable of a reversible swelling-deswelling process in solvents, especially in water. In fact, solid hydrogels in water absorb a considerable amount of liquid and this results in a swelling of the macromolecular structure that becomes soft and translucent. In this form, the hydrogel is well suited to soft tissues and can come in contact with body fluids without causing damage. Thus, any drug molecule present in the hydrogel matrix can be conveyed in biological fluids and carry out his function [8,9,10,11]. In recent years, many formulations of new hydrogels are being studied to search for the best solution to the specific problem [12,13,14]. Recently, we have proposed some polyelectrolyte hydrogels based on vinyl polymers bearing α-amino acid residues [6,7,15]. These platforms, which include the l-phenylalanine, the l-valine and the l-histidine, are well suited to the complexation of ionic/ionizable drugs through the functional groups (carboxyl, aminic nitrogen) present in the polymeric structure. Therefore, these polymers become sensitive to pH changes especially in the range of acidity which includes their pKa. Each pH-stimulation which leads to ionization of the hydrogel promotes its swelling and this can be monitored also by the ionic strength [15]. Moreover, the presence of the amide group further promotes the hydration of the network. On the other hand, the temperature variation may or may not promote its swelling. It is known that any increase in temperature weakens the hydrogen bonds between the water molecule and the amido group to make the poly(*N*-alkyl-acrylamide) polymers insoluble, such as the poly(*N*-isopropylacrylamide) with a lower ‘critical solution temperature’ (LCST) of 32 °C [16,17]. So, the proposed hydrogels can be considered as ‘intelligent hydrogels’ and appropriately applied to carry out their function according to need. In previous papers, we reported the usefulness of these polymers as complexing agents of metal-based drugs (cisplatin, ruthenium compounds) [7,15,18,19,20,21,22,23] and drugs for uses in solid tumor therapy [24], ophthalmology [25], and, not least, for mood disorders [26]. In general, the sustained release of these drugs from the drug loaded-hydrogel was attributed to the different strength of the drug–polymer interaction. As a rule, a higher drug–polymer interaction is highlighted by high pKa values. The formation of a stable adduct ensures a slower release of the drug molecule that may also be prolonged for a long time while keeping the stability of the drug itself. Based on previous results, and especially to strengthen the study of the controlled-release of antidepressant drugs [26], in this paper we report some results on the behavior of two molecules containing secondary amino nitrogens that are used in clinical practice: paroxetine and duloxetine [27,28]. Depression is a disease that affects hundreds of millions of people worldwide, causing nearly one million deaths each year. It was estimated that in 2020 depression will be the second leading cause of work disability because of job loss, poverty and the disease itself [29]. However, with the correct diagnosis and therapy with specific drugs, the recovery of patients could almost always be possible. Paroxetine is an antidepressant drug belonging to the class of SSRIs (selective serotonin reuptake inhibitors), marketed since 1992. Like other SSRIs, its intake leads to an increase of synaptic availability of serotonin neurotransmitter that is deficient in people suffering from depression. Compared to other molecules of the same therapeutic class (fluoxetine, sertraline, etc.) in the same dosage it has a more powerful effect [30]. It is commonly used to treat depression, in panic attacks with or without agoraphobia, in obsessive-compulsive disorder, social phobia cases and in anxiety disorders. Like other drugs of its class, paroxetine is generally preferred to tricyclic antidepressants for its better tolerability and the reduced presence of side effects, especially in terms of cardiotoxicity [31,32]. Duloxetine is an antidepressant that belongs to the class of inhibitors of the reuptake of both serotonin and norepinephrine (SNRI). It possesses an aryl-aryl propyl amine structure that is the basis of not tricyclic antidepressants. Duloxetine is indicated for the treatment of major depressive episodes and diabetic peripheral neuropathic pain in adults. Among the side effects there are anxiety, headache, fever, drowsiness, constipation, abdominal pain, hallucinations, seizures, gastroenteritis, hot flashes, disorders of sexual desire, the presence of vesicles. In 2008, the Food and Drug Administration (FDA) approved a new indication for duloxetine, allowing its use in the treatment of fibromyalgia in adults [33]. A controlled release of the drug from purposely loaded hydrogel can avoid the inconvenience of an initial overdose and reduce the undesirable side effects. Furthermore, the complexed drug in the hydrogel maintains its activity for longer times.

Basically, these two drugs contain in the molecular structure a secondary amino nitrogen of piperidine-type for paroxetine and a secondary amino nitrogen of alkyl chain for duloxetine. Table 1 shows the structure of the two drugs with the respective values of the pKa [34]. In the same table, the acidity constants of two correlated drugs (trazodone and citalopram) bearing tertiary nitrogen atoms [26] and the monomers used for the hydrogel preparation are reported for comparison [35]. As a rule, the basicity of secondary amine nitrogens is greater than the corresponding tertiary amino nitrogens [36]. A comparison of the molecular structures paroxetine-trazodone and duloxetine-citalopram shows a clear higher basicity for paroxetine, while duloxetine has a similar value of pKa with respect to citalopram. It is evident that the pKa values will influence the loading and the consequent release of the drug on the basis of molecular structure and the basicity of the hydrogel ionizable groups. In this study, we will examine the comparison of the paroxetine and duloxetine release by two hydrogels containing l-valine (AVA-5) [25] and l-phenylalanine (PHE-Nip3) [24] moieties in two different aqueous solutions (PBS, pH 7.40; acetate buffer, pH 4.60) at a temperature of 25 °C. Furthermore, for the hydrogel PHE-Nip3 containing embedded magnetic nanoparticles [24], an external alternating magnetic field (AMF) will be applied to trigger different amounts of released drug. A pulsatile controlled drug delivery was characterized for the paroxetine at two different pH values and with remote control of the AMF trigger [37]. The two pH values (pH 4.6 and pH 7.4) were chosen as the corresponding limit values, respectively, of complete collapse and swelling of PHE-Nip3 hydrogel. This behavior can be useful for therapeutic applications involving pH changes in the human body, as for example in the stomach and in the intestine.

## 2. Results and Discussion

### 2.1. Protonation Study

In a molecule, the protonation of the secondary amino nitrogen is stronger than that of the corresponding tertiary amino nitrogen. This is generally established by the acidity constant values (pKa) [36]. Of course, inductive and solvation effects of particular molecules with basic/acid character may cause protonation mechanisms involving values of pKa not conforming to the general rule.

**Figure 1:**
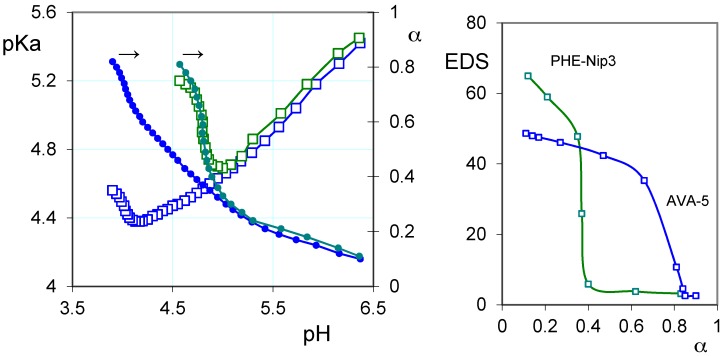
(**Left**) Relationships between the acidity constants (pKa, open squares), degree of protonation (α, filled circles) and pH of the hydrogels PHE-Nip3 (green line) and AVA-5 (blue line); (**Right**) The equilibrium degree of swelling (EDS) in relation to α for the hydrogel PHE-Nip3 (green line) and AVA-5 (blue line) (all data are referred to 25 °C and 0.15 M NaCl).

On the other hand, the protonation thermodynamic study of the hydrogels is the most suited for understanding the drug–polymer stability. Various types of polymers are used as carriers of drugs, linked covalently or ionically through appropriate functional groups [38,39,40]. In the case of ionic interaction, the polyelectrolyte can exert a valid activity for the controlled release of drugs. Such ionic polymers may be subject to conformational changes in which the macromolecule assumes a different extension according to its charge density to form a skein in a completely neutral form [41]. Then, the pH plays a key role in the coil-to-globule transition, especially in the pH-range that includes the polymer pKa. Moreover, the presence of hydrophobic groups on the macromolecule can facilitate the macromolecular collapse at a pH different from that expected. So, it may happen that the polymer collapses at a critical degree of protonation (α) if the hydrophobic attractive forces exceed the repulsive electrostatic forces. This is the case of the two polyelectrolytes containing the l-phenylalanine and the l-valine residues, whose protonation mechanism has highlighted a characteristic thermodynamic behavior [42,43]. The polymers show a critical α in which the macromolecule collapses before even reaching its state of complete neutralization. This behavior is also shown in the corresponding hydrogels, despite the cross-linking of macromolecules [7,15]. This can be of considerable interest since it helps in the design of ionic hydrogels with the prediction of their possible collapse at specific pH values. In the left panel of Figure 1, the characteristic behavior of two hydrogels containing the l-valine and l-phenylalanine residue is shown. These clearly show their intrinsic polyelectrolyte behavior; the values of the acidity constant (pKa) depend on both α and the ionic strength [7]. The same Figure 1 (right panel) shows the equilibrium degree of swelling *EDS* = (*W*_w_ − *W*_d_)/*W*_d_ (where *W*_w_ and *W*_d_ is the weight of wet and dry hydrogel, respectively) for the two hydrogels at different α values.

In any case, the pKa values are ‘apparent’; that is they depend on the degree of protonation, and thus on the pH, showing a decreasing trend up to a critical α value [7]. Above this value, the pKa increases, despite the reduced number of COO^−^ groups present on the polymer [42]. In these conditions, however, the macromolecular collapse exposes a higher concentration of ionic groups causing greater electrostatic effects and, consequently, an ease proton uptake. The network collapse at the critical value of α is due to the opposing hydrophobic–hydrophilic forces. The completely ionized hydrogel gradually reduces its swelling during the protonation, for the reduced number of charged groups; at the same time, the hydrophobic groups (isopropyl and phenyl) develop their attractive force to overcome the electrostatic repulsion forces, causing the collapse at the critical α value. The latter can be controlled by the ionic strength, since the presence of the simple salt, by reducing the shielding effect of the COO^−^ groups, increases both values of pKa and critical α. Even the presence of non-ionic units, such as *N*-isopropylacrylamide (Nip), exerts a significant shielding effect. Its presence in the hydrogel PHE-Nip3, in addition to decreasing the pKa for reduced electrostatic effects of the macromolecule, makes the polymer collapsible to lower α values, and then pH values greater than that observed for the AVA hydrogels. The thermodynamic data are reflected in the swelling phenomenon [7,15] since it shows that the EDS value undergoes a sharp decrease in correspondence of α values closer to 0.4 and 0.7 (pH values 5 and 4), respectively, for the hydrogels PHE-Nip3 and AVA-5. For the two hydrogels AVA-2 and AVA-5, the crosslinking degree only involves a greater effect of swelling, at pH above the critical one, for a lower crosslinking [7]; virtually no variation of pH to the critical point is observed due to the similarity of pKa for the two cases.

### 2.2. Loading of Paroxetine and Duloxetine

The loading of the two drugs was carried out in an aqueous medium on both hydrogels in the fully ionized form. In these conditions, the free hydrogels PHE-Nip3 and AVA-5 in double-distilled water show an EDS of 702 and 194, respectively. The lower EDS value of the hydrogel AVA-5 is attributed to the higher degree of crosslinking, despite the increased number of COO^−^ groups present on the polymer. The loading of drug was obtained by soaking the hydrogels in a concentrated drug solution. The physical contact of the latter immediately led to a considerable reduction of the hydrogel swelling, causing it to collapse to a small volume in the form of dispersed particles. The measured value of EDS for the hydrogel PHE-Nip3 was found to be 4 and 2, respectively, with paroxetine and duloxetine. The hydrogel AVA-5, instead, showed slightly greater values of EDS: 8 with paroxetine and 11 with duloxetine. Furthermore, it was observed that the considerable reduction of the swollen hydrogel was accompanied by a drastic decrease of the concentration of the drug in the contact solution, within the first 5 min of charging. As a result, the hydrogel PHE-Nip3 showed 26.5 wt % of loaded paroxetine and 22.1 wt % of duloxetine. An amount about two times greater of loaded drugs was obtained with the hydrogel AVA-5 because of the increased number of COO^−^ groups: 53.6 wt % (paroxetine) and 44.2 wt % (duloxetine). For the same hydrogel, the greater lightweight load value of the paroxetine is in line with its higher molecular weight. It is evident that the macromolecular collapse led to neutralization of the COO^−^ groups with the consequent loss of the hydration water molecules that held the swollen polymer network. This collapse is at most facilitated by the interaction of the hydrophobic groups present in the monomer units [42,43] which are overwhelming on any electrostatic repulsion of charged residues groups (see Figure 1).

### 2.3. Release of Paroxetine and Duloxetine from the Hydrogels PHE-Nip3 and AVA-5

**Figure 2:**
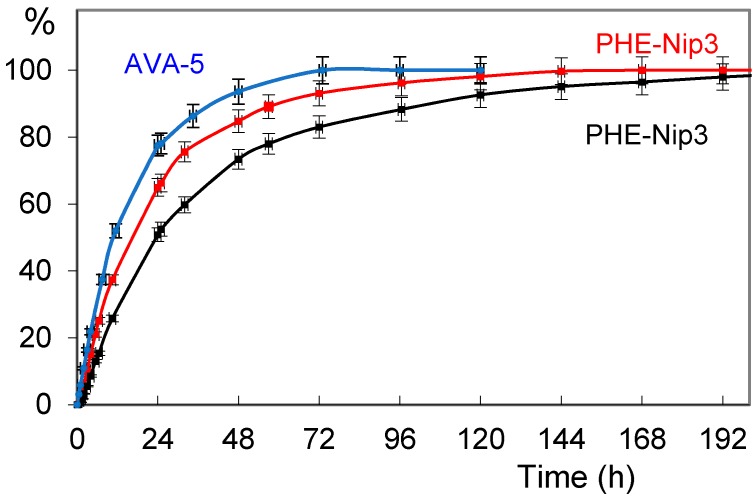
Releasing profiles (% in relation to time) of paroxetine from the hydrogels AVA-5 (blue circles) and PHE-Nip3 without (black squares) and with (red squares) alternating magnetic fields (AMF) stimulation in phosphate buffered saline (PBS) buffer pH 7.40 and 25 °C.

The releasing study of paroxetine and duloxetine from the drug-loaded hydrogels was conducted in aqueous media at 25 °C. The release was triggered by pH changes and by applying an alternating magnetic field of low frequency. In physiological saline solution (PBS pH 7.40), the drug is released from the hydrogel according the kinetic profile shown in Figure 2. The figure shows the release of the paroxetine from the hydrogel AVA-5 and PHE-Nip3, this last showing a slower transport mechanism and in longer times. In fact, unlike the AVA-5 hydrogel that completely downloaded paroxetine in about three days, the hydrogel PHE-Nip3 prolongs its release for times longer than a week. The application of a remote stimulation with an alternating magnetic field (20 kHz and 50 W) produces a considerable increase of paroxetine released during the first five days from the hydrogel PHE-Nip3 and containing embedded cobalt ferrite (CoFe_2_O_4_) magnetic nanoparticles.

Similar behavior was observed for duloxetine. When the hydrogel PHE-Nip3 was considered, the two drugs showed a similar release profile, with a greater release of duloxetine staying within 10% for a long period of time. The analysis of the release curves with the empirical Peppas’s power law expression [44,45]: *M_t_*/*M*° = *kt^n^* (where *M_t_* and *M*° is the cumulative drug released at time *t* and infinite time, respectively; *n* is the diffusion exponent characteristic of the release mechanism; *k* is the rate constant relative to the properties of the matrix and the drug) shows an anomalous non-Fickian transport for paroxetine and duloxetine (Table 2).

In fact, a value of *n* < 1 is associated with a drug transport mechanism not purely of Fickian diffusion. This suggests that a further process, in addition to that of diffusion, must be considered. This process can be associated with the contextual swelling of the hydrogel which increases during the release of the drug. In addition, the drug–polymer interaction exerts a role in establishing the transport mechanism. A stronger interaction, especially electrostatic, leads to a slower release which is reflected in a low value of the rate constant *k* (Table 2). In fact, the release of paroxetine through the hydrogel PHE-Nip3 shows the lowest value of *k* as an index of greater drug–polymer interaction and this value is very similar to those previously reported for the release of citalopram [26]. On the other hand, the release of duloxetine from the hydrogel PHE-Nip3 shows a value of *k* much higher and comparable to that shown for the AVA-5 hydrogel. The application of an alternating magnetic field, in general, leads to an increase of the drug-release from hydrogels containing embedded magnetic nanoparticles (CoFe_2_O_4_). Stimulation of magnetic nanoparticles allows a further stress that promotes the swelling of the hydrogel and the consequent release of the drug. This is clearly shown in Figure 2 for the release of paroxetine from the hydrogel PHE-Nip3. In the first 24 h of release, the magnetic stimulation increases of about 20% the amount of drug available in the saline solution. At the end of the release assessment, the results of measured EDS values are 23.3 and 26.3, respectively, for the samples of hydrogel releasing paroxetine and duloxetine. The small differences in EDS are in line with a greater hydration of the hydrogel due to the greater loss of duloxetine. As reported in Table 2, the effect of the alternating magnetic field causes a general increase of the parameters *k* and *n*, as an index of an improvement in the transport mechanism of the drug through the hydrogel. In some cases, the transport mechanism for paroxetine becomes a Super Case-II transport, with *n* > 1. This is in line with the results previously reported studying different drugs for different purposes, such as doxorubicin [24,26]. When the AMF trigger is applied, the rate of drug release is enhanced for an extended time. The release curve is systematically higher than the control. The energy induced by AMF can cause oscillation or vibration of the network-embedded CoFe_2_O_4_ magnetic nanoparticles [46]. This in turn may cause twisting and/or displacement of the polymeric chains, resulting in an enhancement of the diffusion process. It is believed that the magnetically-induced deformation of the hydrogel as a result of the oscillation or vibration of the embedded magnetic nanoparticles is elastic. This elastic deformation ensures a long-term, reliable controlled release of the drug, as also addressed by other researchers [47,48,49].

A closer behavior of the two drugs was observed for the release profiles from AVA-5 hydrogel. In this case the macromolecular structure displays a greater amount of ionized carboxyl groups, along with a corresponding number of hydrophobic isopropyl groups present in the l-valine moiety. In addition, the hydrogel contains a greater degree of cross-linking (5 mol %) with EBA units making it less swellable. The greater number of carboxyl groups makes it available to a greater loading amount of drugs. In fact, a loading of 53.6 wt % and 44.2 wt % was obtained for paroxetine and duloxetine, respectively. The release of duloxetine and paroxetine from the hydrogel AVA-5 is always greater than that shown by the PHE-Nip3 hydrogel, despite the higher degree of crosslinking. It is to be expected that the increased crosslinking of the polymer ensures a slower release of the drugs. This is in line with the hydrogel AVA-5, which released over time the two drugs. As previously reported, the analogous hydrogel AVA-2 (crosslinked with 2 mol % of EBA), containing uploaded citalopram and trazodone [26], released the drugs in much less time (within 24 h), despite the lower degree of crosslinking and comparable pKa values [7]. This means that, by considering the same drug, the electrostatic interaction between the drug and the hydrogel is weaker for AVA-5, and then the drug is released more rapidly in aqueous saline solution. The greater degree of crosslinking exerts the further role of slowing down the diffusion of the drug through the denser network. In all cases, however, the release of the drug with secondary amino nitrogen is ensured for long periods of time.

#### 2.3.1. Release of the Drug: Effect of pH and Alternating Magnetic Field (AMF)

**Figure 3:**
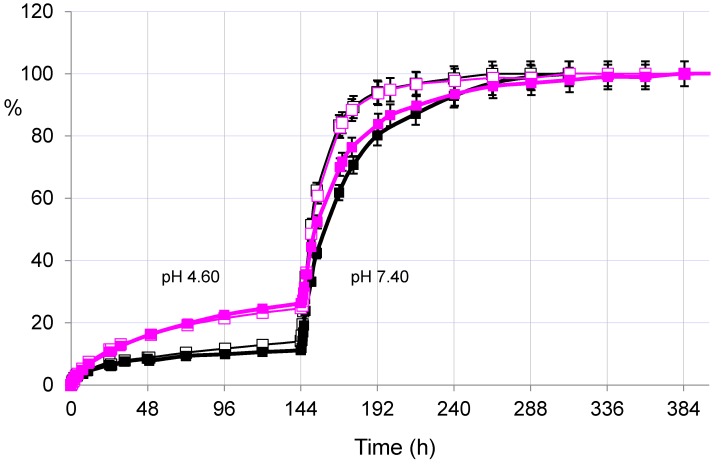
Releasing profiles (% in relation to time) of paroxetine (dark lines) and duloxetine (pink lines) from the hydrogel PHE-Nip3 with (empty squares) and without (filled squares) application of the alternating magnetic field (20 kHz/50 W). The release was performed at 25 °C in acetate (pH 4.60) and in PBS (pH 7.40) buffer solutions.

On the other hand, the evaluation of the release in acetate buffer at pH 4.60 showed once again the significant differences between the two drugs released from the same hydrogel PHE-Nip3. Figure 3 shows a flatter release of paroxetine in the first six days of experimentation at pH 4.60. In the same conditions, the duloxetine experiences an increased and increasing release, comparable to the profile previously reported for trazodone and citalopram [26].

Even the AMF effect seems to have a negligible influence due to poor or absent swellability of the hydrogel at pH lower than its pKa. Only with the change of the external solution at pH > pKa (PBS, pH 7.40) the release, for both drugs and under AMF, reproduces the results reported above: paroxetine and duloxetine are released faster and for a long time under magnetic stimulation.

On the other hand, the release of the drugs from the hydrogel AVA-5 was successively evaluated in the two solutions at pH 4.60 and 7.40. At the lowest value of pH, the amount of released drug was found to be approximately only 10% within the six days of testing time. The increased cross-linking causes a greater reduction of the release, when compared to the previous hydrogel AVA-2 releasing citalopram and trazodone [26].

#### 2.3.2. Pulsatile Drug Release

**Figure 4:**
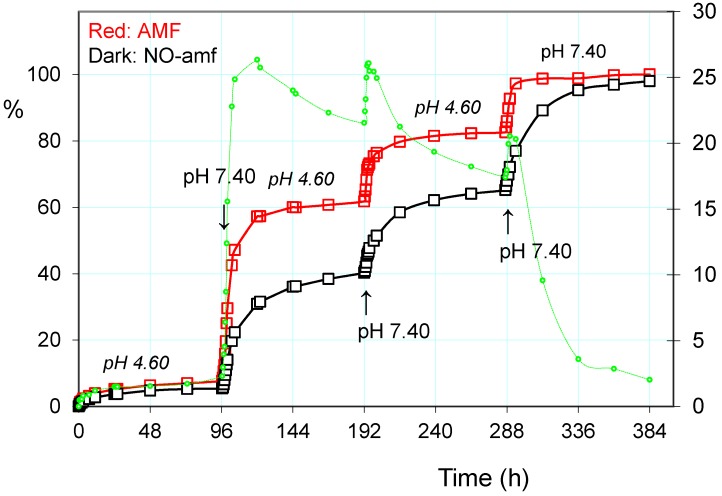
Paroxetine release (%) from the hydrogel PHE-Nip3 on pulsed variation of buffer solutions (PBS, pH 7.40; acetate, pH 4.60) with (red line) and without (dark line) AMF application (20 kHz/50 W). The green dashed line represents the difference (%) between the release values with and without the application of AMF.

Based on the above observations, and mainly of the fast response to stimulation of pH and the alternating magnetic field, the cumulative release of paroxetine from the drug-loaded PHE-Nip3 hydrogel was evaluated at lower pH, under applied pulses at pH 7.40. In particular, a slow and gradual release at pH 4.60 was carried out for a long period of time in which only about 20% of the drug was released (Figure 4). After four days, the outside PBS saline solution at pH 7.40 was maintained only for two hours. In this short period, the greater pH triggered the release that quickly increased to reach values of about four times higher than the previous one. The restoration of pH 4.60 solution did not block the release immediately, but the drug continued to flow in the external solution for a further three hours and then settle the release at low constant values. A second change in the external solution to pH 7.40 showed a further increase of the release rate in the two-hour experience. The subsequent restoration of pH 4.60 solution reported a flat release for a long time of four days. The third and final change in the external PBS solution was constantly maintained throughout the final period of time and up to the completeness of the release of paroxetine. The comparison of the three trigger steps shows lower release of the drug in the second stage than in the first one; its cumulative release goes through about 40% in the first to about 65% in the second stage. The third stage instead recovers most amount of drug as the external solution remains at higher pH 7.40 for all the settled time of four days.

**Figure 5:**
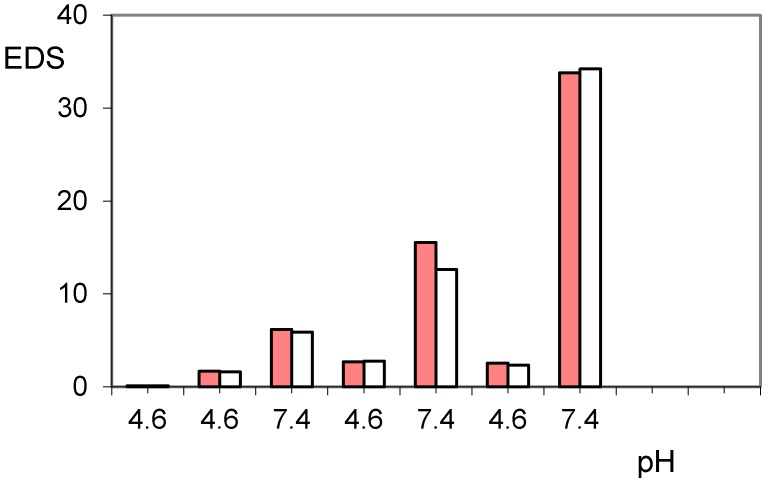
Equilibrium degree of swelling (EDS) of hydrogels PHE-Nip3 after releasing paroxetine at pulsed variations of buffer solutions (acetate, pH 4.60; PBS, pH 7.40) with (red bars) and without (white bars) AMF application.

The addition of a further trigger based on the alternating magnetic field greatly increases the release of paroxetine, especially when the pH pulses are implemented [37]. In the same Figure 4 are reported the three steps of pulses with PBS solution at pH 7.40, superimposed on the normal release at pH 4.60. As is noted at the end of the first pulse stage, there is a net gain of about 20% of drug release; the application of the AMF trigger improves the leakage of the drug. The cumulative drug release becomes less and less pronounced after each pulse of pH, as the remaining amount of loaded drug becomes increasingly lower and this decreases the concentration gradient for the release of the drug itself. It is evident that the unloading of the drug consequently permits hydration of the hydrogel, especially at a pH greater than its pKa. This was measured in terms of EDS that actually reveals a gradual increase in EDS with the repeated pulsation cycles at pH 7.40 (Figure 5). The observed gradualness seems to be not dependent on the effect of AMF, also because the latter produces effects of collapse of the hydrogel due to the developed hyperthermia.

**Figure 6:**
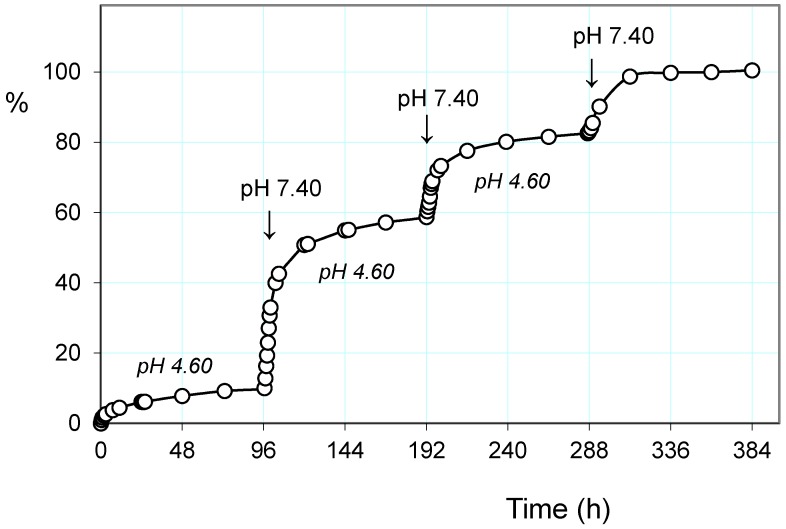
Paroxetine release (%) from the hydrogel AVA-5 on pulsed variation of buffer solutions (PBS, pH 7.40; acetate, pH 4.60) at 25 °C.

On the other hand, also the hydrogel AVA-5 shows a similar behavior to the pulsed release of pH. Figure 6 shows, similarly to above, the release of paroxetine in acetate buffer pH 4.60 and pulsed with three cycles of PBS at pH 7.40.

The trend shows a significantly higher drug release, both at pH 4.60 and pH 7.40, throughout the experimental procedure. This is in agreement with the weaker drug–polymer electrostatic interaction, having the polymer a lower basicity. It is interesting to observe how in this case the values of EDS at the various pH 7.40 pulsation cycles remain fairly constant at a value of about 15 while at pH 4.60 they are significantly higher than those reported for the previous hydrogel PHE-Nip3. This can be attributed to the higher hydration capacity for the greater ionization, as the hydrogel AVA-5 collapses at pH 4.0 (see Figure 1).

## 3. Conclusions

The use of polyelectrolyte hydrogels showing complexing ability towards ionic/ionizable molecules can have a special potential in drug delivery. The loading of the drug is improved by electrostatic effects exerted by the polymer that at the same time preserves its stability [24]. Consequently, the kinetics of the release is controlled, besides the Fickian diffusion, by the drug–polymer interaction forces of the adduct. The stability of the latter depends essentially on the relative strength of the acid-base sites involved. A higher basicity stabilizes the adduct and, consequently, the drug is released slowly and for a longer period of time. The vinyl hydrogels bearing α-amino acids (l-phenylalanine, l-valine) residues are well suited for drug delivery applications. The presence of the carboxyl group of the polymer gets a platform sensitive to changes in pH, especially in the range that includes its pKa. Furthermore, these groups allow the formation of stable adducts with molecules bearing basic sites of secondary amino groups, such as paroxetine and duloxetine. The results of the sustained release of the two drugs used in mood disorder clearly demonstrate the utility of the use of the hydrogels proposed for a more long-term treatment and with the possibility of monitoring ‘on demand’ the amount of extra drug. The release of paroxetine from the two hydrogels AVA-5 and PHE-Nip3 showed how the change in pH of the environment in contact with the hydrogel is also significant for a short time and in repeated pulses. This modular release is possible because of the ionization and consequent swelling of the hydrogel. Then, the free drug is conveyed in the solution more quickly from the swollen networks. Moreover, the presence of embedded magnetic nanoparticles in the hydrogel PHE-Nip3 allows a further remote control when an alternating magnetic field (AMF) is applied [24,37]. The low frequency oscillating magnetic field application relies on interactions between magnetic CoFe_2_O_4_ nanoparticles and resulting mechanical deformation of the hydrogel to squeeze out the drug [50]. Then, on the basis of the experimental results, we can conclude that the proposed biocompatible polyelectrolyte hydrogels can be appropriately used for the design of new pharmaceutical formulations for a long-term therapy of depression. This work supports the hypothesis of using magnetic nanoparticles embedded in polyelectrolyte hydrogels for controlled release of substances and offers a promising technology for “switchable” drug release in the biomedical studies by using an external magnetic field to displace the nanocomposite at designated places within the body [47,48,49,50].

## 4. Experimental Section

### 4.1. Materials and Methods

Buffer solution at pH 4.60 was freshly prepared with milli-Q water (Millipore, Billerica, MA, USA) with a 0.01 mol/L acetic acid (Fluka, Steinheim, Germany) in 0.15 mol/L NaCl. PBS solution at pH 7.40 was prepared by dissolving weighed amounts of salts (from Sigma-Aldrich, St. Louis, MO, USA) NaCl (8.0 g), KCl (0.2 g), K_2_HPO_4_ (1.15 g), and NaH_2_PO_4_ (0.2 g) in 1000 mL of milli-Q water. Paroxetine and duloxetine (cymbalta) were supplied as 20 mg and 60 mg tablets by the EG S.p.A. (Milan, Italy) and Eli Lilly (Indianapolis, IN, USA), respectively. The active ingredient present in the tablet was separated from the other ingredients by dissolving it in methanol (50 mL). The solution was then filtered and brought to dryness. The purity of the obtained compound was determined in aqueous solution by potentiometric acid-base titration. Hydrogels AVA-5 and PHE-Nip3 were prepared according to the synthetic procedure previously reported [24,25]. AVA-5 is a vinyl polymer consisting of *N*-acryloyl-l-valine units and crosslinked with 5 mol % of *N*,*N′*-ethylene-bisacrylamide (EBA). PHE-Nip3 instead is a copolymer consisting of *N*-acryloyl-l-phenylalanine and *N*-isopropylacrylamide units (in a molar ratio 1:1) and crosslinked with 2 mol % of EBA; the cross-linking reaction was carried out in the presence of cobalt ferrite CoFe_2_O_4_ magnetic nanoparticles. Potentiometric titration curves were automatically recorded with a Windows-based software (TimTalk 9, http://www.labsoft.dk/timtalk9.htm) supporting the TitraLab 90 (Radiometer Analytical, Lyon, France) titration equipment [51]. A combined glass pH electrode (Red Rod, Loveland, CO, USA) and a temperature sensor (T201, U.S. Sensor, Orange, CA, USA) were immersed into the 0.15 M NaCl aqueous solution containing a weighed amount of drug and an excess of measured quantity of hydrochloric acid. The solution was maintained in a thermostatted glass cell (50 mL) with stirring and under a presaturated nitrogen stream. Titrations with a sodium hydroxide solution (0.06 M) were performed with an equilibration time of 240 s for each 0.04 mL of titrant addition. The amount of weighed white powder of crude drug in the hydrochloride form was in the range of 20–30 mg. The results of the two measurement were quite in agreement, providing a potentiometric purity greater than 70 wt %. The AMF measurements were supported by an AG 1006 amplifier/generator (T&C Power Conversion, Inc., Rochester, NY, USA) which monitored at given values of 50 W and 20 kHz, the power and the frequency, respectively. The solenoid winding with honeycomb cell was described previously [24]. The spectrophotometric measurements, equipped with 10 mm quartz cuvettes, were conducted on the Specord 210 spectrophotometer (Analytik Jena, Jena, Germany). 

### 4.2. Loading of the Drug

The loading of the two hydrogels, PHE-Nip3 and AVA-5, with the drugs paroxetine and duloxetine was carried out in aqueous media according to the following procedure. Concerning the loading of paroxetine, a weighed amount of dry hydrogel (81.5 mg of PHE-Nip3 and 94.0 mg of AVA-5) was placed to swell in 50 mL distilled water and containing a stoichiometric amount of sodium hydroxide. When the complete swelling was reached (about 1 week), the hydrogel was repeatedly washed with distilled water for a further week by changing the water twice a day. Simultaneously, 200 mg of crude paroxetine hydrochloride product (70 wt % potentiometric purity), obtained by the purification of six EG 20 mg tablets, were dissolved in 150 mL distilled water for the whole night (2.5 × 10^−3^ mol/L). Therefore, the drug solution was filtered and added rapidly to the swollen hydrogel PHE-Nip3 (volume about 60 mL). The rapid collapse of the hydrogel, which occurred within minutes, showed the actual drug loading. This was further verified by the strong decrease of the absorbance values measured at 294 nm [52] which showed a remaining amount of drug in solution (1.3 × 10^−3^ mol/L). In all cases, the evaluation of the amount of paroxetine in solution was made on the basis of a previously recorded calibration curve (Absorbance (*Abs*) = 1960·*C* − 0.0046, where *C* is the drug concentration in mol/L) by taking an aliquot (100 µL) of solution overlying the hydrogel under loading and diluting it to 3 mL with distilled water before measuring the value of the absorbance. The residual solution of paroxetine (1.3 × 10^−3^ mol/L) was subsequently used for the AVA-5 loading. Also in this case the hydrogel collapse occurred in few minutes and the measured absorbance of the final solution was negligible, only pointing out traces of paroxetine (corresponding to about 2 mg). Then, the two paroxetine-loaded hydrogels were filtered on a Strainer cell (BD Falcon, Biosciences, Bedford, MA, USA) and quickly washed with distilled water for several times. Subsequently they were placed to dry in air for about a week. The dry weight provided a brown powder of paroxetine-loaded PHE-Nip3 of 111.0 mg (26.5 wt %) and white granules of paroxetine-loaded AVA-5 of 202.2 mg (53.6 wt %). In a similar manner, the loading of the duloxetine was carried out on two samples of hydrogels (PHE-Nip3, 74.0 mg; AVA-5, 102.7 mg) swollen like the previous ones. Even in these cases, the addition of duloxetine led to a quick collapse of the hydrogel. The amount of drug in each solution was evaluated by spectrophotometric measurements at 290 nm by considering the molar absorption coefficient (ε = 208 mL·mg^−1^·cm^−1^) [53]. The final products of duloxetine-loaded PHE-Nip3 and duloxetine-loaded AVA-5 provided a brown powder and white granules amounting to 95.0 mg (22.1 wt %) and 184.0 (44.2 wt %), respectively. 

### 4.3. Release of the Drug

The two hydrogels loaded with paroxetine and duloxetine were studied in two different buffer solutions at pH 4.60 (acetate buffer, 0.01 mol/L in 0.15 M NaCl) and pH 7.40 (PBS) for the release of the drug. The in vitro release by the hydrogel AVA-5 was conducted in a glass cell, thermostatted at 25 °C and containing the appropriate buffer (50 mL) and a weighed amount of the drug-loaded hydrogel in a Strainer cell (pore size, 70 µm), under magnetic stirring. The weighed amount of the sample ranged within 6–15 mg, with a maximum of three replicates. The amount of drug in solution was monitored at time intervals and determined by spectrophotometric measurements at 294 nm and 290 nm for paroxetine and duloxetine, respectively. When it was needed to change to a different pH buffer solution, the Strainer cell, containing the hydrogel sample, was wiped with tissue paper to remove excess of water and introduced into the new buffer solution (50 mL) to continue the release experiment. The same was conducted for the drug-loaded PHE-Nip3 hydrogel. In this case, however, a parallel control solution, containing about the same amount of sample, has been subjected to stimulation of the alternating magnetic field (AMF, 20 kHz and 50 W) controlled by the AG 1006 amplifier/generator (MDM Electronic, Wrocław, Poland). The plastic container (50 mL), containing the Strainer cell with the weighed sample, was surrounded by a copper coil honeycomb cell. At the end of each experiment, and particularly to the exchange of buffer solutions of different pH, the wet hydrogel was weighed to calculate the value of *EDS* = (*W*_w_ − *W*_d_)/*W*_d_, where *W*_w_ is the weight of wet gel and *W*_d_ is the weight of dry gel. 

## Figures and Tables

**Table 1:** Acidity constants of ionizable antidepressant drugs and monomers.

Chemical Structure	pKa	Chemical Structure	pKa
*Paroxetine*	9.77 ^a^	*Duloxetine*	9.7 ^a^
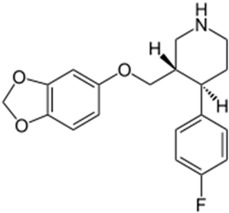		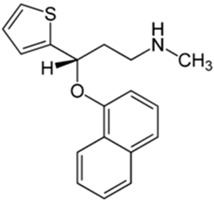	
*Trazodone*	6.89 ^b^	*Citalopram*	9.42 ^b^
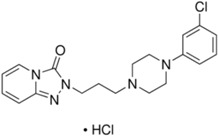		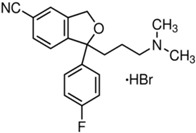	
*N-acryloyl-l-phenylalanine*	3.34 ^c^	*N-acryloyl-l-valine*	3.47 ^c^
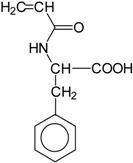		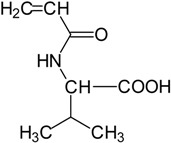	

Source of data: ^a^ [34]; ^b^ [26]; ^c^ [35].

**Table 2:** Parameters of the empirical Peppas’s power law expression (*M_t_*/*M*° = *kt^n^*) for the release of paroxetine and duloxetine through the hydrogels PHE-Nip3 and AVA-5 in PBS buffer (pH 7.40) with and without AMF stimulations.

Hydrogel	Drug	AMF ^a^	*k*	*n*	*R*^2 b^
PHE-Nip3	Parox	N	0.0246	0.96	0.993
		Y	0.0247	1.26	0.995
	Dulox	N	0.0572	0.84	0.990
		Y	0.0594	0.93	0.990
AVA-5	Parox	-	0.0593	0.91	0.998
	Dulox	-	0.0591	1.04	0.996

^a^ Alternating Magnetic Field: 20 kHz, 50 W; ^b^ Correlation coefficient.

## Abbreviations

The following abbreviations are used in this manuscript:

AMF	Alternating magnetic field
EDS	Equilibrium degree of swelling
